# Impact of time of flight and point spread function on quantitative parameters of lung lesions in ^18^F-FDG PET/CT

**DOI:** 10.1186/s12880-021-00699-w

**Published:** 2021-11-13

**Authors:** Kemin Huang, Yanlin Feng, Weitang Liang, Lin Li

**Affiliations:** Department of Nuclear Medicine, The First People’s Hospital of Foshan, Foshan, 528000 Guangdong China

**Keywords:** Tomography, Emission computer, Time of flight, Point spread function, Quantitative parameters, Standard uptake value

## Abstract

**Background:**

Image reconstruction algorithm is one of the important factors affecting the quantitative parameters of PET/CT. The purpose of this study was to investigate the effects of time of flight (TOF) and point spread function (PSF) on quantitative parameters of lung lesions in ^18^F-FDG PET/CT.

**Methods:**

This retrospective study evaluated 60 lung lesions in 39 patients who had undergone ^18^F-fluoro-deoxy-glucose (FDG) PET/CT. All lesions larger than 10 mm in diameter were included in the study. The PET data were reconstructed with a baseline ordered-subsets expectation–maximization (OSEM) algorithm, OSEM + PSF, OSEM + TOF and OSEM + TOF + PSF respectively. The differences of maximum standard uptake value (SUVmax), mean standard uptake value (SUVmean), metabolic tumor volume (MTV), total lesion glycolysis (TLG)and signal to noise ratio (SNR)were compared among different reconstruction algorithms.

**Results:**

Compared with OSEM reconstruction, using OSEM + TOF + PSF increased SUVmean and SUVmax by 23.73% and 22.71% respectively, and SNR increased by 70.18%, MTV decreased by 23.84% (*p* < 0.01). The percentage difference was significantly higher in smaller lesions (diameter 10–22 mm) than in larger lesions (diameter 23–44 mm), and significantly higher in low contrast lesions (SNR ≤ 15.31) than in high contrast lesions (SNR > 15.31). The difference of TLG among various reconstruction algorithms is relatively small, the highest value is − 6.48% of OSEM + TOF + PSF, and the lowest value is 0.81% of OSEM + TOF.

**Conclusion:**

TOF and PSF significantly affected the quantitative parameters of lung lesions in ^18^F-FDG PET/CT. OSEM + TOF + PSF can significantly increased SUVmax, SUVmean and SNR, and significantly reduce MTV, especially in small lesions and low contrast lesions. TLG can be relatively stable in different reconstruction algorithms.

## Background

The application of ^18^F-2-fluoro-2-deoxyglucose (FDG) positron emission computed tomography (PET/CT) hybrid imaging provides diagnostic information for the staging, differential diagnosis, treatment planning and response monitoring of various tumors. Among these applications, SUVmax is the most commonly used quantitative measurement index [1]. However, it is easy to be affected by reconstruction parameters and image statistical noise, and leading to poor consistency of repeated tests compared with other indicators [2, 3]. Recent studies have shown that volume based PET/CT parameters, such as total lesion glycolysis (TLG) and metabolic tumor volume (MTV), may better reflect the burden of the whole tumor compared with SUVmax, and have potential value in clinical staging and treatment monitoring [4–6].

In recent years, PET imaging has undergone important changes with the introduction and development of time of flight (TOF) [7] and point spread function (PSF) [8]. TOF and PSF can improve the spatial resolution and signal-to-noise ratio of images, which may affect the detectability of small and low-intensity lesions [9, 10]. However, compared with the images reconstructed by the conventional ordered subset expectation maximization algorithm, the PET images obtained by these corrections often highlight the image contrast of specific lesion size and cause SUV fluctuation [11, 12]. This uncertainty of SUV by TOF and PSF may affect the apparent size, shape, and radiation uptake of tumors.

One of the main uncertainty factors of PET quantitative analysis is closely related to image reconstruction algorithm and reconstruction parameters. The purpose of this study was to investigate the effects of TOF and PSF on the quantitative parameters of lung lesions such as SUV, MTV and TLG in ^18^F-FDG PET/CT imaging.

## Materials and methods

### Patient population

This retrospective study was approved by our hospital institutional review board, and the informed consent from each patient was obtained. The study population included 39 lung cancer or malignant tumor with lung metastasis patients who underwent ^18^F-FDG PET/CT. Inclusion criteria were: histologically proven cancer with a potential for primary or secondary lung involvement; and one or more pulmonary solid lesions on the CT image. Exclusion criteria were: known inflammatory or infectious lung diseases. Considering the influence of partial volume effect, the lesions larger than 10 mm in diameter in CT images were targeted. In order to reduce the effect of breathing on SUV of lung lesions, the cases with poor registration of CT and PET images were excluded. Table 1 shows the characteristics of the enrolled patients.

**Table 1:** Patient characteristics

Characteristics	Value
Age in years, mean (range)	61.72 (42–81)
*Sex*	
Male (%)	20 (51.28%)
Female (%)	19 (48.72%)
Blood sugar (mmol/l), mean	5.26 (4.2–6.7)
Weight in kg, mean (range)	58.45 (44.0–81.0)
Injected dose in MBq, mean (range)	215 (156–305)
Maximum tumor diameter in mm, mean (range)	23.1 (10.0–44.0)

The maximum tumor diameter in any direction was measured using a CT image

### Imaging protocol and data reconstruction

The PET scanner used in this study was a Siemens Biograph mCT with 64 slice CT(Siemens Medical Solutions, Erlangen, Germany), and the scanner has a four-ring extended axial field of view of 21.6 cm. All patients fasted for 6 h prior to the PET/CT so that their blood sugar levels were below 7.2 mmol/l. ^18^F-FDG was injected intravenously according to the body weight of 3.68 MBq/kg, and the patients were required to rest for 1 h. CT acquisition was performed first with the following parameters: tube voltage 120 kV; tube current 220 mA; collimation 64 × 0.6 mm; thickness 3 mm. Subsequently, PET emission acquisition was performed in 3D mode from the skull base to the midthighs, The duration of each bed position was set to 2 min, and the bed overlap was 23%.

PET data were reconstructed using the following reconstruction algorithms: standard 3-D ordinary Poisson ordered subset expectation maximisation (OSEM), OSEM + TOF, OSEM + PSF and OSEM + TOF + PSF. The reconstruction parameters for TOF were 2 iterations and 21 subsets, and those for non TOF were 2 iterations and 24 subsets. A Gaussian filter with a full width at half maximum of 5.0 mm was used in all reconstruction models. The image matrix was 200 × 200. The pixel size was 3.65 mm. Scatter and attenuation corrections were applied.

### Quantitative measurements

Quantitative measurements were performed on a dedicated workstation (Syngo TrueD, Siemens Medical Solutions).

Volume of interest (VOI) was performed using a 40% threshold of SUVmax. The SUVmax, SUVmean and MTV were obtained for each identified lung lesion. TLG defined as MTV multi-plied by the average SUV uptake (SUVmean) within the MTV. We used the liver as a source for the background and noise measurement [13]. In each patient, a 30 mm-diameter spherical VOI was placed within an area of uniform FDG distribution in the liver, the mean SUV and standard deviation within the VOI were obtained. The signal-to-nose ratio of the tumour, relative to the liver, SNR (T–L) was calculated as:

$$SNR_{(T - L)} = \frac{Tumour - Liver}{{SD_{L} }}$$

where the *Tumour* refers to SUVmax in the lung lesion, *Liver* and *SD*_*L*_ is the mean SUV and standard deviation measured in the liver VOI respectively.

### Data analysis

The relative percentage differences of quantitative parameters of different reconstruction algorithms relative to OSEM were calculated. The lesions were then divided into two groups according to the long-axis diameter of CT images. The median diameter (22 mm) of all lesions was defined as the demarcation line. The first group contained smaller lesions with a diameter 10–22 mm, and the second group contained larger lesions with diameters of 23–44 mm. Besides, all of the lesions were also divided into low contrast group and high contrast group according to the median SNR (15.31). The low contrast group contained lesions with SNR ≦ 15.31, and the high contrast group contained lesions with SNR > 15.31. The differences of quantitative parameters were compared among groups.

### Statistical analysis

All measured values were expressed as mean ± SD, and the relative percentage difference of uptake relative to OSEM were expressed as mean with 95% confidence interval. The comparison of SUVmean and SUVmax between the reconstruction algorithms was performed by the Wilcoxon signed-rank test and using Bonferroni correction. The rank sum test was used to compare the lesions of different size groups and different contrast groups. Pearson regression analysis was used to analyze the correlation between different quantitative parameters. In all analyses, *p* < 0.05 was considered to be statistically significant.

## Results

### Patients

Thirty nine patients [19 women and 20 men; mean age 61.72 years (42–81 years)] met our criteria for participation in the study. A total of 24 lesions in 18 patients with primary lung cancer and 36 lesions in 21 patients with metastatic lung cancer were included in the study. The clinical indications for ^18^F-FDG PET/CT in these patients were lung cancer (n = 18); lung metastasis of colorectal cancer (n = 6); lung metastasis of breast cancer (n = 7); lung metastasis of liver cancer (n = 6); lung metastasis of pancreatic cancer (n = 2). Overall, 60 lung solid lesions larger than 10 mm were detected on CT images. The maximum transverse diameter (long axis) of the lesions was 10–44 mm (median 22 mm). Thirty-two smaller lesions had a maximum transverse diameter 10–22 mm (average 18.9 ± 1.5), 28 larger lesions had a maximum transverse diameter of 23–44 mm (average 28.1 ± 5.9). The SNR of 60 lesions was 2.04–51.95 (median 15.31) in OSEM reconstruction images. Among them, the SNR of 31 low contrast lesions was 2.04–15.31 (average 10.46 ± 3.98), and that of 29 high contrast lesions was 15.32–51.95 (average 27.86 ± 10.67).

### Quantitative PET measurements

**Fig. 1:**
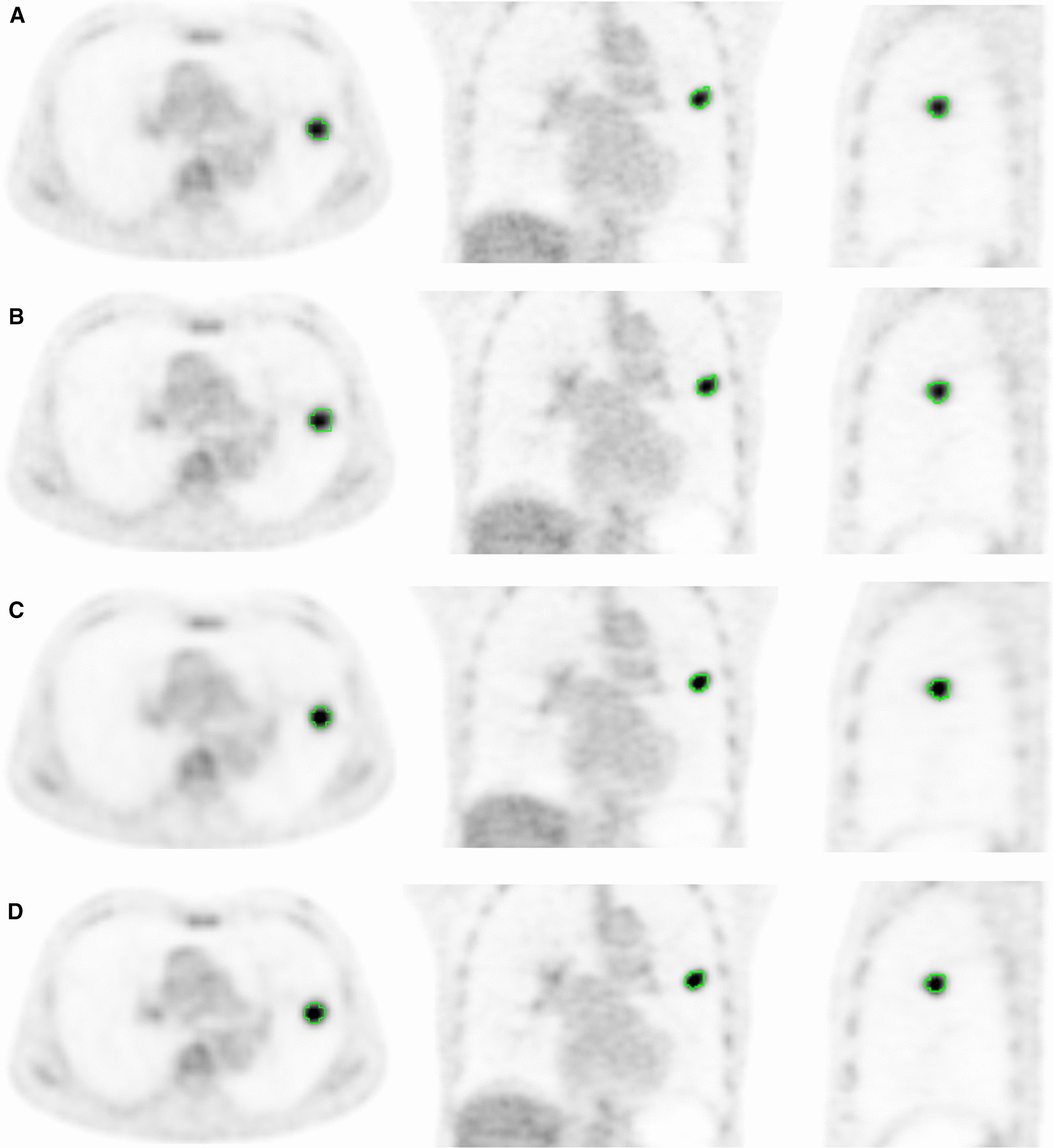
The cross sectional, coronal and sagittal PET images of a male lung cancer patient with different reconstruction algorithms, that the VOI of lung lesions was automatically delineated by 40% threshold of SUVmax, and its quantitative parameters showed significant difference. **A** The OSEM models, SUVmax = 7.22,SUVmean = 4.51, MTV = 2.96, TLG = 13.35; **B** The OSEM + TOF models, SUVmax = 7.42, SUVmean = 4.57, MTV = 2.80, TLG = 12.79; **C** The OSEM + PSF models, SUVmax = 8.88, SUVmean = 5.35, MTV = 2.33, TLG = 12.47; **D** The OSEM + TOF + PSF models, SUVmax = 9.25, SUVmean = 5.59, MTV = 2.20, TLG = 12.30

The quantitative parameters for the lesions as analyzed with each reconstruction algorithm are shown in Table 2. Compared with OSEM reconstruction, when TOF and/or PSF are combined into the image reconstruction, both the SUVmean, SUVmax and SNR of lesions are significantly increased (*p* < 0.05), MTV is significantly decreased (*p* < 0.05); TLG is significantly decreased in OSEM + TOF + PSF and OSEM + PSF (*p* < 0.05), but there is no significant change in OSEM + TOF (*p* > 0.05). Figure 1 shows the VOI examples of different reconstruction methods based on the 40% threshold of SUVmax. It can be seen that the shape and size of the lesions VOI with different reconstruction methods are significantly different.

**Table 2:** Comparison of quantitative PET/CT parameters of different reconstruction algorithms

Reconstruction meoth	SUVmax	SUVmen	MTV	TLG	SNR
OSEM	7.28 ± 2.72	4.36 ± 1.71	2.94 ± 2.15	13.98 ± 15.65	18.87 ± 11.78
OSEM + TOF	7.50 ± 2.66^*^	4.51 ± 1.69^*^	2.85 ± 2.13^*^	14.05 ± 16.00	19.87 ± 11.14^*^
OSEM + PSF	8.34 ± 3.29^**^	5.01 ± 2.05^**^	2.48 ± 1.88^**^	13.22 ± 15.00^**^	28.20 ± 17.66^**^
OSEM + TOF + PSF	8.87 ± 3.18^**^	5.36 ± 2.01^**^	2.31 ± 1.91^**^	13.23 ± 15.71^**^	30.08 ± 15.85^**^

Compared with OSEM, ^**^ P < 0.01, ^*^ P < 0.05

**Fig. 2:**
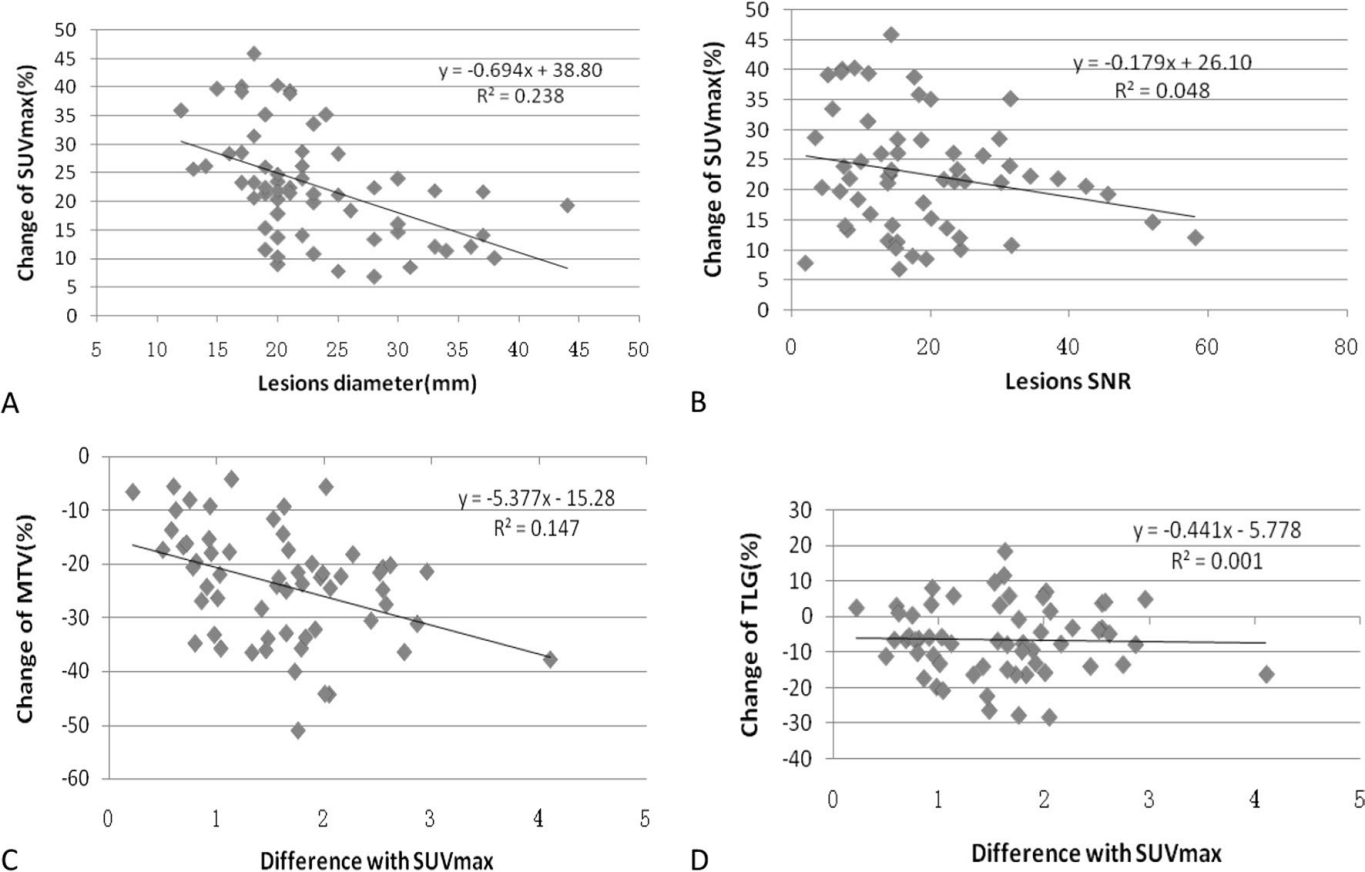
The distributions of the percentage change associated with the SUVmax, MTV and TLG measured by OSEM + TOF + PSF. A total of 60 lung lesions were analyzed. **A** Shows the correlation between percentage change of SUVmax and lesions diameter (F = 18.189, *p* < 0.01). **B** Shows the correlation between percentage change of SUVmax and lesions SNR (F = 2.947, *p* > 0.05). **C** Shows the correlation between percentage change of MTV and difference with SUVmax (F = 10.065, *p* < 0.01). **D** Shows the correlation between percentage change of TLG and difference with SUVmax (F = 0.063, *p* > 0.05)

The percentage changes of quantitative parameters after PSF and TOF reconstruction compared with OSEM are shown in Table 3. The highest value of SUVmean, SUVmax and SNR can be obtained by using OSEM + TOF + PSF, which increased by 22.63%, 23.44% and 69.65% respectively, and the lowest value of MTV was obtained, which decreased by − 23.21%. The percentage changes of TLG obtained by various reconstruction algorithms is relatively small, which the highest value is − 6.48% of OSEM + TO + PSF, and the lowest value is 0.81% of OSEM + TOF. The correlation between different quantitative parameters of OSEM + TOF + PSF are shown in Fig. 2. The percentage differences of SUVmax relative to OSEM was negatively correlated with lesion diameter(r = − 0.489, *p* < 0.001), but there was no significant correlation with the lesion SNR(r = − 0.220, *p* > 0.05); The percentage differences of MTV increased with the change of SUVmax (r = − 0.489, *p* < 0.05), but the TLG was not significantly correlated with the change of SUVmax(r = − 0.033, *p* > 0.05).

**Table 3:** Percentage changes in quantitative PET/CT parameters of TOF and PSF reconstruction relative to OSEM

	OSEM + TOF + PSF			OSEM + PSF			OSEM + TOF		
	Mean	SD	95% CI	Mean	SD	95% CI	Mean	SD	95% CI
Change of SUVmax (%)	22.71	9.64	20.22 to 25.20	13.80	7.10	11.97 to 15.64	4.07	8.15	1.97 to 6.18
Change of SUVmean (%)	23.73	10.51	21.01 to 26.44	13.98	7.84	11.96 to 16.01	4.31	7.21	2.45 to 6.17
Change of MTV (%)	− 23.84	10.51	− 26.55 to − 21.13	− 15.96	10.63	− 18.17 to − 13.22	− 2.78	12.06	− 5.90 to − 0.33
Change of TLG (%)	− 6.48	10.03	− 9.07 to − 3.89	− 4.92	7.23	− 6.79 to − 3.05	0.81	9.24	− 1.57 to 3.20
Change of SNR (%)	70.18	37.21	60.56 to 79.79	50.16	18.12	45.48 to 54.84	9.88	21.65	4.29 to 15.47

### The influence of lesion size

**Fig. 3:**
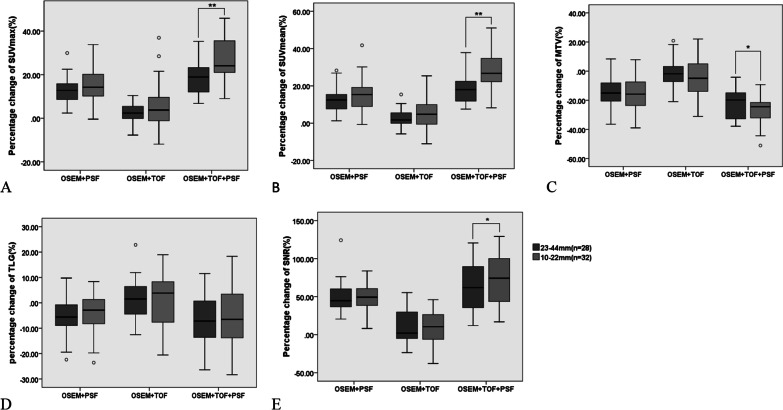
The percentage change of SUVmax (**A**), SUVmean (**B**), MTV (**C**), TLG (**D**), SNR (**E**) of the smaller and larger lesions according to different reconstruction algorithms. The numbers of the two groups were 32 and 28, respectively. OSEM + TOF + PSF provided the higher SUVmax, SUVmean, SNR and lower MTV in smaller lesions than that in larger lesions (**p < 0.01, *p < 0.05)

According to the long axis diameter, the lesions were divided into two groups for further analysis (Fig. 3). For smaller lesions with a diameter of 10–22 mm, the combination of TOF and PSF resulted in a greater changes in SUVmax, SUVmean, MTV and SNR compared with larger lesions with diameters of 23–44 mm (*p* < 0.05). The changes of lesions with diameter of 10–22 mm were 26.39%, 28.44%, − 26.22 and 72.51%, compared with 18.51%, 18.34%, − 21.12 and 67.51% for lesions with diameters of 23–44 mm, respectively. However, there was no significant difference in TLG between large and small lesions (*p* > 0.05), which were − 7.23% and − 5.82% respectively. The changes of SUVmean and SUVmax obtained by TOF and PSF alone were higher in small lesions than those of larger lesions, but the differences were not statistically significant (*p* > 0.05).

### The influence of lesion contrast

**Fig. 4:**
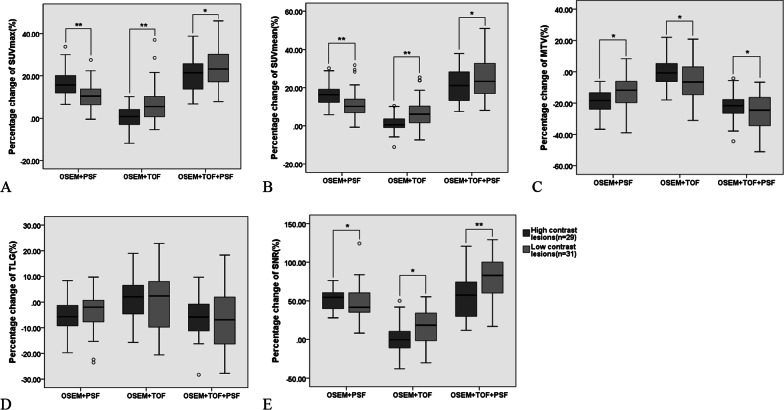
The percentage change of SUVmax (**A**), SUVmean (**B**), MTV (**C**), TLG (**D**), SNR (**E**) of the high contrast and low contrast lesions according to different reconstruction algorithms. The numbers of the two groups were 29 and 31, respectively. OSEM + TOF + PSF provided the higher SUVmax, SUVmean, SNR and lower MTV in low contrast lesions than that in high contrast lesions (**p < 0.01, *p < 0.05)

Besides, we divided the lesions into high contrast and low contrast according to the lesion SNR for further assessment (Fig. 4). In OSEM + TOF + PSF images, the low contrast lesions obtained the higher SUVmax, SUVmean and SNR and lower MTV than those of high contrast lesions (*p* < 0.05). However, there was no significant difference in TLG between high contrast and low contrast lesions (*p* > 0.05). OSEM + PSF had higher SUVmax, SUVmean and SNR in high contrast lesions (*p* < 0.05). However, for the OSEM + TOF images, low contrast lesions showed significantly higher SUVmax, SUVmean and SNR than those of high contrast lesions (*p* < 0.05).

## Discussion

Image reconstruction algorithm is one of the important factors affecting image quality and quantitative accuracy of PET/CT imaging. In recent years, the iterative algorithm based on TOF and PSF has been widely used in clinical imaging, and the image quality has improved significantly [14–16]. This study investigated the effects of TOF and PSF reconstruction on quantitative parameters of lung lesions in ^18^F-FDG PET/CT. According to our results, the effect of TOF and PSF reconstruction on the quantitative parameters of lung lesions is significant, especially for the measurements of SUVmax, SUVmean and MTV. Among them, OSEM + TOF + PSF can significantly increased SUVmax, SUVmean and SNR, and significantly reduce MTV, especially in small lesions and low contrast lesions; The effect of TOF and PSF on TLG was relatively small, These results indicate that TLG can more stably express the metabolic state of ^18^F-FDG in lung lesions than other quantitative parameters.

TOF and PSF reconstructions have been proved to improve the signal-to-noise ratio and the detection ability of small lesions. However, some studies [17, 18] have shown that PSF may cause edge artifacts in PET images, which combined with partial volume effect and local statistical noise, have a significant impact on SUVmax. Shang et al. [19] investigated the effect of TOF and PSF on small lesions in integrated PET/MR imaging, and observed that the combination of TOF and PSF increased the SUVmean by 26.6% and the SUVmax by 30.0%. Akamatsu et al. [20] analyzed 41 lymph node metastases, and found that OSEM + TOF + PSF increased the SUVmax by 43.3% and the SUVmean by 31.6% compared with conventional OSEM. In our study, compared with OSEM reconstruction, OSEM + TOF + PSF obtained the largest difference, with SUVmean and SUVmax increased by 22.63% and 23.44% respectively. However, the difference was relatively small when using OSEM + TOF reconstruction alone, with the SUVmean and SUVmax increased by 4.31% and 4.07% respectively.

Previous studies have shown that there is a negative correlation between lesion diameters and the degree of increase in SUV after PSF reconstruction[20–22], although the results did not always reach statistical significance. Lasnon et al. [23] reported that compared with 1 cm or greater lesions, the increase of SUVmax, SUVmean and lesion/background ratio using PSF reconstruction were more marked in lesions less than 1 cm. Our study found that there was a significant negative correlation between the degree of increase in SUV and lesion diameter after OSEM + TOF + PSF reconstruction. The changes of SUVmax and SUVmean were 26.39% and 28.44% in 10–22 mm lesions, which were significantly greater than 18.51% and 18.34% of 23–44 mm lesions. Besides, the increase of SUV in larger lesions was higher than that in smaller lesions after PSF or TOF reconstruction alone, but the difference was not statistically significant, which may be related to the fact that we only analyzed the lesions larger than 10 mm.

In the study of Rogasch et al. [24], the effect of TOF and PSF on SUV was related to the lesion background ratio, TOF provided the largest increase of SUVmax in low contrast lesions, whereas PSF showed a related increase in high contrast lesions. Our results are consistent with previous studies. The increase of SUV after OSEM + TOF + PSF and OSEM + PSF reconstruction was larger in low contrast lesions than in high contrast lesions, while OSEM + TOF was larger in high contrast lesions than in low contrast lesions. Therefore, it should be realized that the specific effects of PSF or TOF on PET/CT quantitative parameters are also affected by different lesion size and lesion background ratio. Finally, in clinical practice, we should choose the appropriate reconstruction algorithm according to the actual situation, and keep in mind the dependence of SUV on reconstruction method, lesion size and lesion contrast. When comparing different PET/CT scan results, the consistency of reconstruction methods and reconstruction parameters should be ensured. At the same time, a standardized protocol should be established to ensure the comparability of quantitative parameters between different PET scans, especially in therapeutic response assessment.

The MTV is an indicator of metabolic activity using tumor volume. It was reported that the use of MTV might reflect tumor prognosis more precisely than SUVmax [25, 26]. There are several methods available to measure tumor volume using PET images. For example, relative threshold method and gradient segmentation method. The relative threshold method refers to the method of counting 30%, 40% or 50% of voxels of the SUVmax value [27, 28] to obtain tumor volume, it is expected that the overestimation of the SUV correlates with the increase in threshold value, and consequently, if the SUVmax value is overestimated, the measured tumor volume may be underestimated.

In a previous study, the PSF reconstruction significantly increased SUVmax and significantly underestimated MTV when using gradient segmentation method to delineate tumor volume [29]. In our study, the MTV of OSEM + TOF + PSF was significantly lower compared with OSEM reconstruction, and the percentage change was negatively correlated with the change of SUVmax. Simultaneously, the degree of MTV reduction was greater in smaller lesions than in larger lesions, and greater in low contrast lesions than in high contrast lesions. Therefore, if TOF and PSF images are used for tumor volume measurement, the threshold level of each reconstruction model should be recalculated according to the fluctuation of SUV, and the influence of lesion size and lesion contrast should be considered.

TLG defined as MTV multiplied by the average SUV uptake (SUVmean) within the MTV, which is an index of the neoplastic cell density [30]. TLG provides important benefits in staging, in tailoring therapeutic strategies, and in outcome prediction of solid tumors [31]. However, volume-based indices were suggested to be influenced by partial volume effect, the segmentation method, and the reconstruction algorithm of the PET scan [31, 32]. The actual deviation of volumetric parameters of ^18^F-FDG-PET, including MTV and TLG, after TOF and PSF reconstruction has not been well discussed in the literature. In the study on lung tumors patients by Armstrong et al. y[33] suggested that the percentage changes of TLG were less than 10% after the combination of TOF and PSF reconstruction, but with large variations in the amount of changes.

Our results are consistent with previous studies. The difference of TLG in different reconstruction algorithms is relatively small, and there is no significant difference in different size and contrast lesions. This result may also be related to the definition of TLG as MTV times SUVmean. For the fixed threshold VOI delineation method, when the SUVmax increases, the MTV measurement value will inevitably decrease, and concurrently lead to the increase of SUVmean, thus reducing the influence of the change of SUVmax on TLG and keeping it relatively stable. In this study, the percentage differences of MTV increased with the change of SUVmax, but there was no significant correlation between the percentage change of TLG and the change of SUVmax, which is the reflection of this result. In conclusion, TLG may be less sensitive to reconstruction methods, lesion size and contrast than SUVmax, SUVmean and MTV, and has a small relative difference compared with OSEM, indicating that TLG is a relatively robust uptake measure and may be more suitable for tumor staging and prognosis than SUVmax, but large sample clinical practice and more evidence foundation are needed.

The limitations of this study include relatively small number of participants, and the limitations of specific patient population such as weight range. Besides, in order to minimize the impact of partial volume effect, we limited the lesions to those larger than 10 mm, and there may be differences for lesions smaller than 10 mm. In this study, we focused on the effects of different reconstruction algorithms on PET quantitative parameters. There was no histological correlation between FDG uptake measured in the lesions, so it is not possible to determine which reconstruction algorithm can reflect the true metabolic activity of the lesion. Further research is needed to address this limitation. Secondly, we only evaluated lung lesions, and the effect of reconstruction algorithm on other parts of the body may have different performance. The data of this study are only applicable to specific scanner.

## Conclusion

This study investigated the effects of TOF and PSF reconstruction on quantitative parameters of lung lesions in ^18^F-FDG PET/CT. The measured quantitative parameters fluctuate obviously among different reconstruction algorithms. TOF and PSF reconstruction can significantly increased SUVmax, SUVmean and SNR, and significantly reduce MTV, especially in small lesions and low contrast lesions. TLG can be relatively stable in different reconstruction algorithms, different sizes and contrast of lesions, which suggests that TLG may be a more suitable quantitative parameter than SUVmax, especially in the monitoring of treatment response.

## Data Availability

The datasets used or analysed during the current study are available from the corresponding author on reasonable request.
